# Risk Factors and Outcomes of Preterm Premature Rupture of Membranes in a Cohort of 6968 Pregnant Women Prospectively Recruited

**DOI:** 10.3390/jcm8111987

**Published:** 2019-11-15

**Authors:** Damien Bouvier, Jean-Claude Forest, Loïc Blanchon, Emmanuel Bujold, Bruno Pereira, Nathalie Bernard, Denis Gallot, Vincent Sapin, Yves Giguère

**Affiliations:** 1 Biochemistry and Molecular Genetic Department, CHU Clermont-Ferrand, Faculty of Medicine, Université Clermont-Auvergne, CNRS 6293, INSERM 1103, GReD, 63000 Clermont-Ferrand, France; vsapin@chu-clermontferrand.fr; 2 Department of Molecular Biology, Medical biochemistry and Pathology, Faculty of Medicine, Centre de recherche du CHU de Québec-Université Laval, Québec City, QC G1V 0A6, Canada; jean-claude.forest@chudequebec.ca (J.-C.F.); yves.giguere@crchudequebec.ulaval.ca (Y.G.); 3 Faculty of Medicine, Université Clermont-Auvergne, CNRS 6293, INSERM 1103, GReD, 63000 Clermont-Ferrand, France; loic.blanchon@uca.fr; 4 Department of Obstetrics, Gynecology and Reproduction, Faculty of Medicine, Université Laval, Québec City, QC G1V 0A6, Canada; Emmanuel.bujold@crchudequebec.ulaval.ca; 5 Biostatistics Unit (DRCI), CHU de Clermont-Ferrand, 63000 Clermont-Ferrand, France; bpereira@chu-clermontferrand.fr; 6 Centre de recherche du CHU de Québec-Université Laval, Québec City, QC G1L 3L5, Canada; nathalie.bernard@crchudequebec.ulaval.ca; 7 Department of Obstetrics and Gynecology, CHU Clermont-Ferrand, Faculty of Medicine, Université Clermont-Auvergne, CNRS 6293, INSERM 1103, GReD, 63000 Clermont-Ferrand, France; dgallot@chu-clermontferrand.fr

**Keywords:** PPROM, risk factors, outcomes, gestational diabetes

## Abstract

We revisited risk factors and outcomes related to the preterm premature rupture of membranes (PPROM). A total of 7866 pregnant women were recruited during 5 years at their first prenatal visit to the perinatal clinic of the institution. We compared three groups (women without prematurity, women with spontaneous preterm labor with intact membranes (sPL with IM), women with PPROM) regarding 60 criteria about characteristics, lifestyle, medical, gynecological, obstetrical history of mothers, medication during pregnancy, events at delivery, and complications in neonates. Logistic regression analyses adjusting for potential confounding factors were used. Of the 6968 women selected, 189 (2.8%) presented a PPROM, and 225 (3.2%) an sPL with IM. The specific risk factors for PPROM were body mass index (BMI) <18.5 kg/m^2^ (adjusted odds ratio, aOR: 2.00 (1.09–3.67)), history of PPROM (aOR: 2.75 (1.19–6.36)), nulliparity (aOR: 2.52 (1.77–3.60)), gestational diabetes (aOR: 1.87 (1.16–2.99)), and low level of education (aOR: 2.39 (1.20–4.78)). The complications associated with PPROM were abruption placentae, cesarean, APGAR 5′ <4, birth weight <2500 g, stillbirth, neonatal jaundice, and hospitalization of mother and neonates. All these complications were also associated with sPL with IM. Our study confirms some of the risk factors of PPROM and highlights a new one: gestational diabetes. Outcomes of PPROM are related to prematurity.

## 1. Introduction

Preterm premature rupture of membranes (PPROM), defined as rupture of fetal membranes prior to 37 weeks of gestation, complicates approximately 2%–4% of all pregnancies and is responsible for 40% of all spontaneous preterm births [1,2], while spontaneous preterm labor with intact membranes (sPL with IM) represents 60% of spontaneous preterm births [3]. PPROM arises from complex pathophysiological pathways that include inflammation and oxidative stress [4]. Although many factors can increase the risk of PPROM, its cause is not fully understood [5]. Among the socio-behavioral and demographic risk factors of PPROM are poor socio-economic status and low level of education, smoking, difficult working conditions, and African ethnicity [6,7]. Other factors have been proposed, such as maternal age and increased or decreased body mass index (BMI) [6,8,9]. Also, a history of PPROM, a history of prematurity, or multiple pregnancies are predominant considered risk factors [2,10,11]. Other factors, such as nulliparity, the interval between pregnancies (<6 or >60 months), cervico-isthmic abnormalities, genital infections, and hydramnios, have also been reported [8,10,12,13]. The outcomes associated with PPROM include prematurity, oligohydramnios, abruption placentae, intrauterine infection, and chorioamnionitis [2,14,15]. 

Most studies on risk factors and outcomes of PPROM have been heterogeneous with regards to methodologies and the number of characteristics investigated, and only a few were prospective large-scale longitudinal cohorts. We proposed to revisit the risk factors and outcomes of PPROM, based on a well-characterized large-scale prospective cohort of 7866 pregnant women recruited at their first prenatal visit to the perinatal clinic of the institution. The important collection of data during the constitution of this cohort allowed the study of many criteria related to PPROM. To distinguish between factors specific to PPROM and those that are rather related to prematurity itself, this cohort was subdivided into three groups: women without prematurity (delivery >37 weeks of gestation), women presenting sPL with IM, and women with PPROM.

## 2. Experimental Section

### 2.1. Study Design

**Figure 1:**
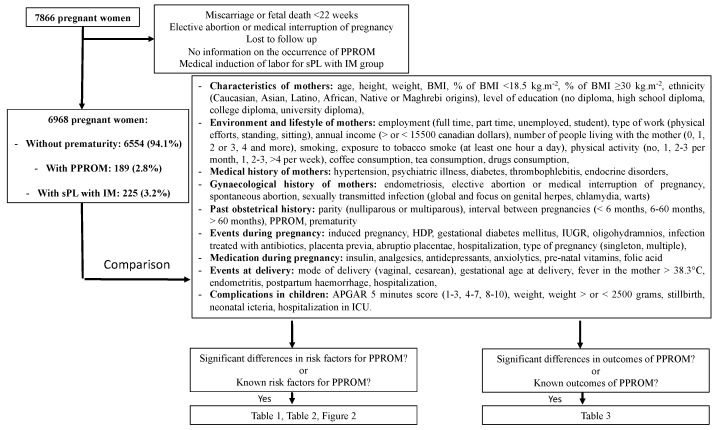
Flowchart of the study. BMI: Body mass index, HDP: Hypertensive disorders of pregnancy, ICU: Intensive care unit, IUGR: Intrauterine growth retardation, PPROM: Preterm premature rupture of membranes, sPL with IM: spontaneous preterm labor with intact membranes.

This study was based on a large prospective cohort of 7866 pregnant women recruited at the “CHU de Québec-Université Laval” from April 2005 to March 2010 at their first prenatal visit to the perinatal clinic of the institution. Follow-up visits were all performed within the institution by a research nurse. Pregnant women aged 18 years or older were eligible to participate in the study. Exclusion criteria were pregnant women without information on the occurrence of PPROM (yes or no), pregnancies termination (elective abortion or medical interruption of pregnancy), miscarriage or fetal death before 22 weeks of gestation, failed to follow-up, and medical induction of labor for sPL with IM group (Figure 1). Participants gave written informed consent, and the study was approved by the “CHU de Québec” Ethics Review Board (initial approval date: 9 November 2004, Project 5-04-10-01 (95.05.17l SC12-01-159). 

The diagnosis of PPROM was confirmed by well-established clinical and/or biological diagnostic procedures: the visualization of amniotic fluid passing from the cervical canal and pooling in the vagina, a basic pH test of vaginal fluid, or arborization (ferning) of dried vaginal fluid identified under microscopic evaluation [16]. The comparisons between 3 groups (delivery >37 weeks, sPL with IM, PPROM) on 60 selected criteria focused on characteristics of the study participants, their environment and lifestyle, medical, gynecological and obstetrical history, events and medication during pregnancy, events at delivery, and complications in neonates (Figure 1). Diagnosis of hypertensive disorders of pregnancy (HDP) was made by a senior obstetrician according to the classification of the Society of Obstetricians and Gynecologists of Canada based on information retrieved from medical records, which includes gestational hypertension (GH) and preeclampsia. GH was defined as de novo hypertension (systolic blood pressure ≥140 mmHg and/or diastolic blood pressure ≥90 mmHg) after 20 weeks of pregnancy. Preeclampsia was defined as GH with proteinuria (≥300 mg in a 24-h urine collection or ≥2+ on the dipstick in a random sample) or pre-existing hypertension and new or worsening proteinuria. Gestational diabetes mellitus (GDM) diagnosis was established according to the Canadian Diabetes Association 2013 Clinical Practice Guidelines [17]. In agreement with these recommendations, most women (90.7%) had a 50 g glucose challenge test (GCT) between 24 and 28 weeks of gestation, followed by a 75 g oral glucose tolerance test (OGTT) if the result of the GCT was between 7.8 and 10.2 mmol/L. GDM was diagnosed if the result of the GCT was ≥10.3 mmol/L or if one or more values equaled or exceeded the thresholds of 5.3, 10.6, and 9 mmol/L at 0, 1, and 2 h, respectively, during the OGTT. Infections treated with antibiotics included genital infections, urinary tract infection, ear, nose, and throat infections, and mouth infections. 

### 2.2. Statistical Analysis

All statistical analyses were performed using Stata statistical software (version 13, StataCorp, College Station, TX, US). The categorical data were expressed as a number of patients and/or associated percentages. The continuous parameters were expressed as the mean ± standard deviation or median (minimum, maximum, interquartile range), according to a statistical distribution. The assumption of normality was tested using the Shapiro–Wilk’s test. The comparisons between 3 independent groups (delivery >37 weeks of gestation, sPL with IM, PPROM) were performed using a multinomial regression model for categorical parameters and ANOVA or Kruskal–Wallis test when the assumptions of ANOVA were not met (normality and homoscedasticity by the Bartlett test) for continuous variables. Then, multivariable analysis was performed using multinomial (polytomous) logistic regression. The covariates were retained according to univariate results and to their clinical relevance. For risk factors significant in univariate analysis, the adjustment was based on the known risk factors of PPROM: age of mothers, BMI <18.5 kg.m^−2^, history of PPROM, history of prematurity, parity, infection treated with antibiotics, multiple pregnancies, level of education, standing while working, annual income, smoking during pregnancy. Then, attention was given to the study of interactions between significant factors and the presence of multicollinearity. The final model was validated by a two-step bootstrapping process. For each step, bootstrap samples with replacements (*n* = 1000) were generated from the training set. In the first phase, the percentage of models, including each initial variable, was determined by the usual stepwise approach. Then, in the second phase, the parameters of generalized linear regression (multinomial regression for categorical-dependent variable) of the final model were independently estimated. The bootstrap estimates associated with each covariate regression coefficient, and their associated standard errors, were finally averaged from replicates. Log-likelihood measured the goodness-of-fit of a model.

For the study of outcomes, the adjustment was made for the age of mothers, BMI <18.5 kg.m^−2^, smoking during pregnancy, HDP, GDM, infection treated with antibiotics. Results were expressed in regression coefficients (from multiple linear regression) or odds ratios (OR), and then in adjusted OR (aOR) (from logistic regression) and 95% confidence interval. To guaranty the robustness of our results and our conclusions, a sensitivity analysis was carried out to study the statistical nature of missing data (at random or not) and their possible impact, particularly on multivariable analyses. It was confirmed that missing data did not alter multivariable results. A *p*-value of less than 0.05 was considered significant.

## 3. Results

### 3.1. Description of the Cohort

Of the 6968 women selected, 189 (2.8%) presented a PPROM, and 225 (3.2%) presented an sPL with IM (Figure 1). More than 98% of the study subjects were Caucasians. No significant differences (*p* = 0.14) were observed between the mean age of mothers in the control group (30 years (SD: 4.3)) and the PPROM group (29.7 years (SD: 4.7)) or in the sPL group (29.3 years (SD: 4.3)) (Table 1). No significant differences (*p* = 0.21) were observed between pre-pregnancy BMI of mothers in the control group (23 kg.m^−2^ (min: 13.9; max: 58.3: interquartile range, IQR: 20.8–26.6)) and the PPROM group (23 kg.m^−2^ (min: 16.2; max: 50.8; IQR: 20.7–26.5)) or in sPL with IM group (22 kg.m^−2^ (min: 16.2; max: 44; IQR: 20.1–25.4)) (Table 1).

### 3.2. Study of Risk Factors

The significant risk factors for PPROM are presented in Table 1 and Table 2. After adjustment for the known risk factors of PPROM (Table 2), the risk factors for PPROM only were BMI <18.5 kg/m^2^ (aOR: 1.91 (1.04–3.52)), history of PPROM (aOR: 2.61 (1.13–6.03)), nulliparity (aOR: 2.56 (1.77–3.70)), gestational diabetes (aOR: 1.83 (1.14–2.94)), low level of education (aOR: 2.34 (1.08–5.05)), standing while working (aOR: 1.62 (1.04–2.53)), and insulin intake (aOR: 2.51 (1.37–4.59)). The risk factors for both PPROM and sPL with IM were history of prematurity (aOR: 5.23 (2.97–9.22)), multiple pregnancies (aOR: 21.88 (12.45–38.47)), infection treated by antibiotics (aOR: 1.84 (1.26–2.68)), and anxiolytics intake (aOR: 2.45 (1.16–5.18)).

**Figure 2:**
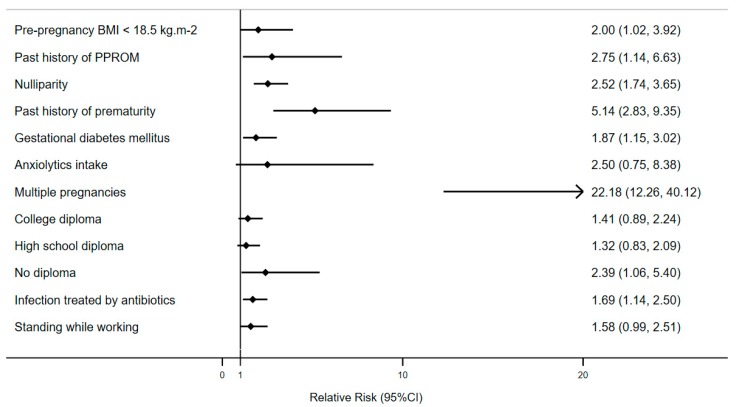
Forest plot showing odds ratios for risk factors of preterm premature rupture of membranes integrated into a multivariable analysis after a bootstrapping process. Horizontal lines represent 95% CIs. CI: confidence interval.

All these risk factors were integrated in a further multivariable analysis (except for insulin intake since it is strongly associated to gestational diabetes), and they all remained significant. The final predictive multivariate model of PPROM included BMI <18.5 kg/m^2^ (aOR: 2.00 (1.09–3.67), *p* = 0.03), history of PPROM (aOR: 2.75 (1.19–6.36), *p* = 0.02), history of prematurity (aOR: 5.14 (2.92–9.06), *p* < 0.0001), nulliparity (aOR: 2.52 (1.77–3.60), *p* < 0.0001), gestational diabetes (aOR: 1.87 (1.16–2.99), *p* = 0.01), infection treated with antibiotics (aOR: 1.69 (1.15–2.47), *p* = 0.007), multiple pregnancies (aOR: 22.18 (12.68–38.81), *p* < 0.0001), low level of education (aOR: 2.39 (1.20–4.78), *p* = 0.01), standing while working (aOR: 1.58 (1.01–2.46), *p* = 0.04), and anxiolytics intake (aOR: 2.50 (1.20–5.24), *p* = 0.02). All these risk factors remained significant at the end of the bootstrapping process except for standing while working and anxiolytics intake (Figure 2).

### 3.3. Study of Outcomes

As expected, the median gestational age at delivery was significantly lower (*p* < 0.0001) in the PPROM group (35.6 weeks (min: 22.6; max: 36.9; IQR: 33.9–36.3)) and in the sPL with IM group (35.9 weeks (min: 22.6; max: 36.9; IQR: 34.3–36.7)) than in the control group (39.7 weeks (min: 37; max: 42.9: IQR: 38.9–40.4))(Table 3). The maternal and neonatal outcomes tested for their potential association with PPROM before and after adjustment are presented in Table 3. After adjustment, the complications associated with PPROM were oligohydramnios (aOR: 4.17 (2.37–7.35)), abruptio placentae (aOR: 4.28 (1.87–9.78)), cesarean (aOR: 1.41 (1.02–1.96)), APGAR 5′ <4 (aOR: 23.32 (7.04–77.19)), birth weight <2500 g (aOR: 47.74 (32.52–70.08)), stillbirth (1.1% in PPROM group versus 0% in control group, *p* < 0.0001), neonatal jaundice (aOR: 3.25 (2.20–4.80)), hospitalization of mother (aOR: 1.75 (1.15–2.65)), and admission at the neonatal intensive care unit (aOR: 17.12 (12.23–23.98)). All these complications were also associated with sPL with IM (Table 3). Birth weight <10th percentile did not increase in both PPROM and sPL with the IM group (Table 3).

## 4. Discussion

Our study confirmed the most known risk factors for PPROM, such as BMI <18.5 kg/m^2^, history of PPROM or prematurity, nulliparity, multiple pregnancies, low level of education, and infections. These results, as well as the percentage of PPROM (2.7%), validated the cohort, which is comparable with others [6,8,11,18,19,20,21]. Our study invalidated, at least in our cohort, other parameters previously found as independent risk factors for PPROM, namely low annual income, smoking, maternal age, increased BMIs, and the interval between pregnancies (<6 months or >60 months) [6,8,10,12,19,22]. It is noteworthy that low annual income, difficult working conditions, and smoking were significantly more prevalent and associated with PPROM before multivariate analysis or bootstrapping process, but not after adjustment. This suggested that these factors might interact with each other and with the level of education, which remained significant. Our study also identified a new risk factor of PPROM, gestational diabetes, which resulted in a 1.87-fold increased risk. This finding was in line with a recent case-control study (one case for one control) of 400 subjects that showed that diabetes mellitus, without distinction between pre-pregnancy diabetes and gestational diabetes, was associated with PPROM [23]. The complications associated with PPROM, such as oligohydramnios, abruption placentae, APGAR 5′ <4, weight <2500 g, stillbirth, neonatal jaundice, and hospitalization of neonates in ICU, are not related to PPROM per se but are associated with prematurity [5,13,24]. A higher proportion of hospitalization of mothers with PPROM contributes to the economic burden of this complication but also increases indirectly the risk of thromboembolism [25]. 

A strength of our prospective study was the homogeneity of our general population. With predominantly Caucasian (>98%) women and a public health system where all pregnant women have access to similar pregnancy follow-up and monitoring, the possibility of sampling bias is reduced. With regard to socio-economic aspects, free access to perinatal care for the Quebec population may limit biases and contributes to the generalizability of the results. The homogenous origin of our cohort can, however, be a limitation since it does not allow measuring the impact of ethnicity, such as African origin, a risk factor of PPROM [7]. Thus, the external validity of our results, especially regarding GDM, should be tested in populations of different ethnic backgrounds. The lack of information on the prolongation of pregnancy after PPROM (latency period) could be considered as a potential limitation. Indeed, a latency period could be associated with a higher incidence of complications [26]. 

Infections, history of prematurity, and multiple pregnancies are known risk factors of both PPROM and also sPL with IM. The association of infection with prematurity at large is in line with recent hypotheses presenting infection in PPROM as a downstream event rather than a causal factor [4]. Recent reports indicated that PPROM might be associated with the presence of sterile inflammation in the fetal membranes [27,28]. Sterile inflammation may be responsible for the link between GDM and PPROM. Indeed, diabetes can promote the production of advanced glycation endproducts (AGEs), ligands of RAGE, a receptor implicated in this pathway [29]. Moreover, a recent study showed that first-trimester AGEs blood levels were significantly higher in cases complicated with PPROM [30]. Criteria related to precariousness, such as BMI <18.5 kg/m^2^ and low level of education, are risk factors of PPROM only. These factors may have a role in the pathology of fetal membranes described by Menon and Richardson by generating oxidative stress [4]. Folic acid supplementation decreased in the PPROM group but did not reach statistical significance in the multivariable analysis. It is to be noted that the fortification of cereal grain products with folic acid could explain the absence of a significant link between PPROM and folic acid in Canada [31]. The oxidative stress, which may be secondary to malnutrition related to precariousness, is another well-established pathophysiological hypothesis of PPROM genesis. Furthermore, the fact that there is no increase in the proportion of birth weight slower than the 10th percentile in the PPROM group, unlike in the sPL with IM group, suggests a pathology of the fetal membranes rather than of the fetus.

## 5. Conclusions

We performed a study of risk factors and outcomes of PPROM, based on a large unselected prospective cohort. Our study confirmed the most risk factors of PPROM, invalidated others (as smoking), and highlighted a new one, GDM, which was associated with a 1.87-fold increased risk. The other specific risk factors for PPROM were BMI <18.5 kg/m^2^, history of PPROM, nulliparity, and low level of education. Outcomes of PPROM are clearly related to prematurity. Further investigations are necessary to understand the pathophysiological link between GDM and PPROM. Combining maternal characteristics and environmental and clinical risk factors to candidate biomarkers—in various large cohorts of pregnant women—may result in proposing a clinically useful predictive model identifying asymptomatic women at higher risk of PPROM. 

## Figures and Tables

**Table 1:** Risk factors of preterm premature rupture of membranes.

	Control group *n* = 6554 (94.1%)	Women with PPROM *n* = 189 (2.8%)	Women with sPL with IM *n* = 225 (3.2%)
**Age and weight of mothers**			
Mean age of mothers (SD) in years	30 (4.3)	29.7 (4.7)	29.3 (4.3)
Median of pre-pregnancy BMI (min, max, IQR) in kg.m^−2^	23 (13.9, 58.3, 20.8–26.6)	23 (16.2, 50.8, 20.7–26.5)	22 (16.2, 44, 20.1–25.4)
Pre-pregnancy BMI <18.5 kg.m^−2^ (%)	5	9.2 *	5.1
**Medical history of mothers**			
History of endometriosis (%)	2.1	4.6 *	6.7 ***
History of PPROM (%)	1	5.4 ***	2.7 *
History of prematurity (%)	4.3	14.1 ***	17.2 ***
Nulliparity (%)	46.7	59.3 **	43.1
**Other complications during pregnancy**			
Hypertensive disorders of pregnancy (%)	4.3	5.8	7.6 *
Gestational diabetes mellitus (%)	6.9	12.2 *	7.1
Infection treated by antibiotics (%)	15.7	26.6 ***	25.5 ***
**Medication during pregnancy**			
Insulin intake (%)	3.4	8.1 **	3.7
Analgesics intake (%)	22.3	31.9 *	33.5 ***
Antidepressants intake (%)	3.2	6.3 *	7.6 **
Anxiolytics intake (%)	1.9	5.7 **	4.6 *
Folic acid supplementation (%)	64.6	55.6 *	58.1
**Multiple pregnancies**			
Multiple pregnancies (%)	0.7	12.2 ***	14.7 ***
**Socio-economic status and lifestyle of the mother**			
No diploma (%)	3.8	7.7 *	5.9
High school diploma (%)	23.5	27.7	26.6
College diploma (%)	32.8	33.6	31.9
University diploma (%)	39.9	31	35.6
Standing while working (%)	35.3	49.5 *	47.5 *
Annual income <15,500 canadian dollars (%)	3.4	7 *	5.1
Smokers during pregnancy (%)	12.3	18 *	18.7 *
Exposure to tobacco smoke (%)	34.6	42.9 *	30.7

BMI: Body mass index; IQR: Interquartile range; PPROM: Preterm premature rupture of membranes; SD: Standard deviation; sPL with IM: spontaneous preterm labor with intact membranes. Significant difference with control group: * *p* < 0.05; ** *p* ≤ 0.001; *** *p* ≤ 0.0001.

**Table 2:** Risk factors of preterm premature rupture of membranes (adjusted odds ratio).

	Women with PPROM *n* = 189 (2.8%)	Women with sPL with IM *n* = 225 (3.2%)
**Weight of mothers**		
Pre-pregnancy BMI <18.5 kg.m^−2^	1.91 (1.04–3.52)	0.78 (0.35–1.74)
**Medical history of mothers**		
History of endometriosis	1.74 (0.74–4.09)	2.92 (1.47–5.82)
History of PPROM	2.61 (1.13–6.03)	0.75 (0.29–1.93)
History of prematurity	5.23 (2.97–9.22)	6.10 (3.93–9.47)
Nulliparity	2.56 (1.77–3.70)	1.09 (0.79–1.52)
**Other complications during pregnancy**		
Hypertensive disorders of pregnancy	1.24 (0.65–2.37)	1.60 (0.91–2.79)
Gestational diabetes mellitus	1.83 (1.14–2.94)	0.97 (0.55–1.70)
Infection treated by antibiotics	1.84 (1.26–2.68)	1.70 (1.20–2.40)
**Medication during pregnancy**		
Insulin intake	2.51 (1.37–4.59)	0.90 (0.40–2.03)
Analgesics intake	1.39 (0.97–1.99)	1.41 (1.02–1.94)
Antidepressants intake	1.66 (0.83–3.32)	2.02 (1.12–3.63)
Anxiolytics intake	2.45 (1.16–5.18)	2.02 (0.96–4.25)
No folic acid supplementation	1.39 (0.96–2.01)	1.17 (0.83–1.64)
**Multiple pregnancies**		
Multiple pregnancies	21.88 (12.45–38.47)	28.86 (17.58–47.38)
**Socio-economic status and lifestyle of the mother**		
No diploma	2.34 (1.08–5.05)	0.94 (0.44–2.04)
High school diploma	1.35 (0.88–2.06)	0.87 (0.60–1.26)
College diploma	1.37 (0.84–2.21)	0.80 (0.52–1.24)
University diploma	1	1
Standing while working	1.62 (1.04–2.53)	1.41 (0.92–2.16)
Annual income <15,500 canadian dollars	1.65 (0.80–3.40)	1.18 (0.56–2.48)
Smokers during pregnancy	1.24 (0.77–2.00)	1.50 (0.98–2.30)
Exposure to tabacco smoke	1.19 (0.81–1.74)	0.65 (0.45–0.95)

BMI: Body mass index; PPROM: Preterm premature rupture of membranes; sPL with IM: spontaneous preterm labor with intact membranes. Adjustment of odds ratio (aOR) was made on the age of mothers, BMI <18.5 kg.m^−^^2^, antecedent of PPROM, the antecedent of prematurity, parity, infection treated by antibiotics, multiple pregnancies, level of education, standing while working, annual income, smokers during pregnancy.

**Table 3:** Outcomes of preterm premature rupture of membranes.

		Control group *n* = 6554 (94.1%)	Women with PPROM *n* = 189 (2.8%)	Women with sPL with IM *n* = 225 (3.2%)
**Implications for pregnancy and childbirth**				
Median gestational age at delivery (min, max, IQR) in weeks		39.7 (37, 42.9, 38.9–40.4)	35.6 (22.6, 36.9, 33.9–36.3) ***	35.9 (22.6, 36.9, 34.3–36.7) ***
Oligohydramnios (%)		2	8.7 ***	5.5 **
*aOR*		*/*	*4.17 (2.37–7.35)*	*2.62 (1.42–4.83)*
Abruptio placentae (%)		0.8	4 ***	3.7 ***
*aOR*		*/*	*4.28 (1.87–9.78)*	*4.68 (2.16–10.11)*
Cesarean (%)		21.3	29.1 *	52.4 ***
*aOR*		*/*	*1.41 (1.02–1.96)*	*4.06 (3.09–5.35)*
**Complications in neonates**				
APGAR 5 min	0 or 1 or 2 or 3 (%)	0.1	2.7 ***	3.2 ***
	*aOR*	*/*	*23.32 (7.04–77.19)*	*30.62 (10.34–90.66)*
	4 or 5 or 6 or 7 (%)	1.5	9.3 ***	11.1 ***
	*aOR*	*/*	*6.67 (3.85–11.54)*	*8.25 (5.12–13.30)*
	8 or 9 or 10 (%)	98.4	88 ***	85.7 ***
	*aOR*	*/*	1	1
Weight in percentile	*<10th (%)*	*5.8*	2.7 *	8.1
	*aOR*	*/*	*0.38 (0.15–0.93)*	*1.37 (0.82–2.27)*
	*10th–90th (%)*	*83.9*	90.9	82
	*aOR*	*/*	*1*	*1*
	*>90th (%)*	*10.3*	6.4	9.9
	*aOR*	*/*	*0.61 (0.34–1.10)*	*1.05 (0.67–1.66)*
Weight <2500 g (%)		1.4	40.6 ***	47.8 ***
*aOR*		*/*	*47.74 (32.52–70.08)*	*69.43 (48.45–99.48)*
Neonatal jaundice (%)		17.2	40.2 ***	32.1 ***
*aOR*		*/*	*3.25 (2.20–4.80)*	*2.31 (1.59–3.36)*
Stillbirth (%)		0	1.1 ***	1.3 ***
**Hospital stay**				
Babies hospitalized in intensive care unit (%)		3.7	41.7 ***	46 ***
*aOR*		*/*	*17.12 (12.23–23.98)*	*21.56 (15.84–29.35)*
Mothers hospitalization (%)		8.9	16.6 ***	22.2 ***
*aOR*		*/*	*1.75 (1.15–2.65)*	*2.64 (1.88–3.72)*

IQR: Interquartile range; (a)OR: (adjusted) odds ratio; PPROM: Preterm premature rupture of membranes; sPL with IM: spontaneous preterm labor with intact membranes. Significant difference with control group: * *p* < 0.05; ** *p* ≤ 0.001; *** *p* ≤ 0.0001. adjustment of OR was made on the age of mothers, body mass index <18.5 kg.m^−2^, smokers during pregnancy, hypertensive disorders of pregnancy, gestational diabetes mellitus, infection treated by antibiotics.
